# A screening program to test and treat for *Helicobacter pylori* infection: Cost-utility analysis by age, sex and ethnicity

**DOI:** 10.1186/s12879-017-2259-2

**Published:** 2017-02-20

**Authors:** Andrea M. Teng, Giorgi Kvizhinadze, Nisha Nair, Melissa McLeod, Nick Wilson, Tony Blakely

**Affiliations:** Department of Public Health, University of Otago, Wellington, New Zealand

**Keywords:** Gastric cancer, Cost-utility analysis, Screening program, Cost-effectiveness, Serology

## Abstract

**Background:**

The World Health Organization recommends all countries consider screening for *H. pylori* to prevent gastric cancer. We therefore aimed to estimate the cost-effectiveness of a *H. pylori* serology-based screening program in New Zealand, a country that includes population groups with relatively high gastric cancer rates.

**Methods:**

A Markov model was developed using life-tables and morbidity data from a national burden of disease study. The modelled screening program reduced the incidence of non-cardia gastric cancer attributable to *H. pylori*, if infection was identified by serology screening, and for the population expected to be reached by the screening program. A health system perspective was taken and detailed individual-level costing data was used.

**Results:**

For adults aged 25–69 years old, nation-wide screening for *H. pylori* was found to have an incremental cost of US$196 million (95% uncertainty interval [95% UI]: $182–$211 million) with health gains of 14,200 QALYs (95% UI: 5,100–26,300). Cost per QALY gained was US$16,500 ($7,600–$38,400) in the total population and 17% (6%-29%) of future gastric cancer cases could be averted with lifetime follow-up. A targeted screening program for Māori only (indigenous population), was more cost-effective at US$8,000 ($3,800–$18,500) per QALY.

**Conclusions:**

This modeling study found that *H. pylori* screening was likely to be cost-effective in this high-income country, particularly for the indigenous population. While further research is needed to help clarify the precise benefits, costs and adverse effects of such screening programs, there seems a reasonable case for policy-makers to give pilot programs consideration, particularly for any population groups with relatively elevated rates of gastric cancer.

**Electronic supplementary material:**

The online version of this article (doi:10.1186/s12879-017-2259-2) contains supplementary material, which is available to authorized users.

## Background

*Helicobacter pylori* is an important contributor to gastric cancer incidence [1]. Globally, the treatment of *H. pylori* has tended to focus on people presenting to medical attention with peptic ulcers and indigestion (dyspepsia). A recent Cochrane systematic review provides evidence that treating people with *H. pylori* infection reduces their subsequent risk of gastric cancer by one- third (95% CI: 0.46-0.95) [2]. In addition to treating *H. pylori* symptomatic individuals, there has been international interest in using *H. pylori* testing to screen asymptomatic individuals in order to prevent gastric cancer from developing. For example, the Asia–Pacific consensus guidelines for *H. pylori* infection recommend screening and treating *H. pylori* infection in communities with high incidence of gastric cancer incidence (20+ per 100,000 population) [3]. Targeted screening has been recommended in some high-income countries for people who are asymptomatic and at high risk, such as having an ethnicity or geographic background associated with a high risk of gastric cancer (e.g. Asia-Pacific country of origin) [4]. The International Agency for Research on Cancer (IARC) and the World Health Organization (WHO) recommends that all countries assess the current and future human and economic impacts of gastric cancer and of the potential value of prevention strategies [5].

In addition to other important social values (such as equity), intervention cost-effectiveness is an important consideration in the prioritization and funding of health services. Ten or more cost-effectiveness evaluations for *H. pylori* screening, such as serology testing, have been published from both high and low gastric cancer incidence countries including China, Taiwan, Singapore, United States, Canada and the United Kingdom. All evaluations found that *H. pylori* screening and treatment was cost-effective, given country-specific willingness-to-pay thresholds [5]. The models tended however to have important limitations [5] including: (i) not using meta-analysis effect sizes for *H. pylori* treatment [2, 6] (although some model estimates were similar to meta-analyses estimates [7–10] and had similar uncertainty [7, 8]); (ii) all but two [9, 11] modelling studies were based on life-years-saved rather than quality adjusted life years (QALYs); (iii) few studies [11–13] conducted a probabilistic analysis to evaluate the uncertainty in the data modeled; (iv) harmful effects of treatment were generally not considered; and (v) there were no data on low-income countries. This paper aims to address the first four of these limitations.

New Zealand is an ideal high-income country for such a case study given the good quality national datasets that support modeling the cost-effectiveness of a *H. pylori* screening program. The age-standardized incidence of gastric cancer for New Zealand in 2011 was 8.0 for men and 4.3 per 100,000 for women and incidence in Māori was 18.6 for men and 14.9 per 100,000 for women (WHO World Standard Population, all ages). Gastric cancer demonstrates one of the greatest ethnic inequalities of any cancer site in New Zealand being 2.48 times higher for Māori (indigenous population) and 2.64 times higher for Pacific peoples compared to NZ European (averaged over 1981-2004) [14]. *H. pylori* infection is likely the greatest contributor to those inequalities [15].

We therefore undertook an assessment of the cost-effectiveness of a *H. pylori* population screening program in New Zealand. The indigenous Māori population was selected to examine the relative cost-effectiveness of screening for a group with higher *H. pylori* infection prevalence and gastric cancer incidence. This selection was appropriate given excellent ethnicity data for Māori in New Zealand, and the New Zealand Government’s treaty and ethical obligations to protect indigenous health.

## Methods

We developed a Markov macrosimulation model to evaluate cost-effectiveness of a *H. pylori* screening program in New Zealand in 2011 compared with current medical practice. A total population screening program for 25-69 year olds was compared with a targeted screening program for Māori 25-69 years old.

### Model overview

**Fig. 1:**
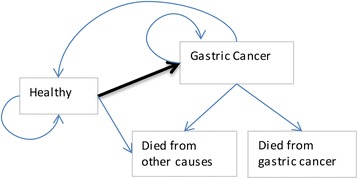
Model structure for the Markov macrosimulation model for studying a *H. pylori* screening interventions to reduce gastric cancer in a population

The Markov model consisted of states including: healthy, gastric cancer, death from gastric cancer and death from other causes (Fig. 1). We did not explicitly model states of *H. pylori* infection and pre-cancer but instead aimed to have a simple model structure and ensure that transition probabilities ‘captured’ different *H. pylori* prevalence in population groups.

### Intervention and comparator definition

The modeled screening programs comprised a serology test to screen for *H. pylori* in healthy individuals, eradication treatment with antibiotics from a primary care doctor if required, a fecal antigen test of cure (retest) and second - line treatment if required. The recommended first-line eradication was triple therapy (omeprazole 20 mg, amoxicillin 1 g and clarithromycin 500 mg twice daily for seven days [16]). Second-line eradication was quadruple therapy [16] and this was not followed by a retest. We assumed that all individuals testing positive would be treated.

Similar to many other cost-utility analyses [7–11, 13, 17–19], we adopted the *H. pylori* serology for the screening test in the main results because of its feasibility, acceptability and affordability. The alternative fecal antigen screening test is a test of active infection, however the test costs more than serology, requires a sample to be delivered promptly to the laboratory and requires patients to stop taking omeprazole, antibiotics and bismuth (OAC) for two to four weeks prior to the test, which is likely to be more problematic for participants. However, we present key results and uncertainty for fecal antigen-based screening in a probabilistic sensitivity analysis. Fecal antigen screening costs were adjusted to reflect differences in the laboratory cost and the fewer positive tests expected, by adopting a screening study finding from Japan, where fecal antigen had consistently 8% fewer positive tests compared to serology (56%/61%) [20]. Fecal antigen was selected in preference to urea breath test, because the former is widely used in this setting and more affordable, despite possible differences in acceptability.

### Input parameters

#### Baseline parameters

The transition rate from ‘healthy’ to ‘gastric cancer’ was governed by cancer registry incidence rates and varied by ethnicity [21]. A 2% decline in gastric cancer incidence over time was incorporated in the model, consistent with global [5, 22] and New Zealand trends. Mortality rates from other causes were obtained from official New Zealand life tables [23], and the rates of gastric cancer death were derived from New Zealand cancer registry [24].

We used a modified version of a traditional quality-adjusted life-year referred to as QALY^DW^. QALYs^DW^ are essentially the same as traditional QALYs, except in two aspects: we use disability weights (rather than utilities) derived using pairwise comparison methods in population surveys from the Global Burden of Disease 2010 study [25], and we allow for sex and age-specific background morbidity from a New Zealand burden of disease study [26]. We use the term QALY^DW^ in the methods sections of this paper, but default to “QALYs” elsewhere.

#### Effect size

A meta-analysis by Ford et al. [2] reported that the reduction in gastric cancer (referred to here as the effect size) among people with *H. pylori* infection who were treated vs not treated was 34% (RR: 0.66, CI: 0.46-0.95). The effect size is only relevant to cases of gastric cancer expected to occur in people with *H. pylori* infection that is detected by screening. We therefore applied the effect size to the number of gastric cancer cases that potentially could be prevented i.e. non-cardia gastric cancer that was attributable to *H. pylori*, for people who are likely to be screened (i.e. coverage) and where *H. pylori* is likely to be detected by the screening test (i.e. sensitivity of the screening test) (Table 1). We used these specifications:.

**Table 1:** Summary of intervention input parameters to the Markov model for a *H. pylori* screening program in New Zealand

	Māori (indigenous population) central estimate (95% CI)/[95% UI]	Non-Māori (rest of NZ population) central estimate (95% CI)/[95% UI]
Effect size		
Meta-analysis rate ratio for incidence of gastric cancer in people with *H. pylori* infection who were treated compared to untreated^b^	Both: 0.66 (0.46–0.95)	
Proportion gastric cancer that was non-cardia gastric cancer (2007-11)		
Men	0.77 (0.69–0.85)	0.45 (0.41–0.49)
Women	0.89 (0.82–0.96)	0.63 (0.58–0.69)
Non-cardia gastric cancer where *H. pylori* infection is detectable^a^	0.89 (0.85–1.00)	
Sensitivity of the serology test (*Se*_*sero*_)	0.89 [0.85–0.92] [28]	
Expected coverage of serology test (using data from routine heart and diabetes checks in NZ adults [27])^a^	0.81 (0.69–0.93)^b^	0.84 (0.72–0.97)^b^
Eradication rate reported by studies in the meta-analysis [2]	0.73 (0.71–0.75)	
Eradication rate of OAC triple therapy in NZ, intention to treat [29]^a^	0.64 (0.53–0.75)	0.86 (0.75–0.96)
Eradication rate of OBTM quadruple therapy in NZ [16]^a^	0.7 [0.6–0.8]	
Annual percentage decline in gastric cancer over time [5]	0.02 [0.01–0.03]	
Screening pathway		
*H. pylori* seroprevalence (proportion) by age-group (years) in 2011 [41]		
25–29	0.11 [0.07–0.14]	0.08 [0.05–0.10]
30–34	0.15 [0.11–0.20]	0.10 [0.07–0.13]
35–39	0.20 [0.14–0.25]	0.13 [0.09–0.16]
40–44	0.24 [0.17–0.31]	0.15 [0.11–0.20]
45–49	0.29 [0.20–0.37]	0.18 [0.12–0.23]
50–54	0.33 [0.23–0.43]	0.20 [0.14–0.26]
55–59	0.38 [0.26–0.49]	0.23 [0.16–0.29]
60–64	0.42 [0.29–0.55]	0.25 [0.18–0.33]
65–69	0.47 [0.33–0.60]	0.28 [0.19–0.36]
Risk of *Clostridium difficile* infection post-treatment (Brown et al., 2013)	0.0008 [0.0004–0.0012]	
Cost of hospitalization with moderate or severe *C. difficile* infection [34]	$3,856 [3085–4628]	
Costs of screening program (NZ, 2011) (See Additional file 1: Table S2 for breakdown of costs and sources)		
Cost per person invited (fixed health promotion, program costs)	$39.87 [31.90–47.84]	
Cost per person tested (test and result)	$54.66 [43.73–65.59]	
Cost per person with a positive test (GP visit, treatment, retest, complications)	$176.70 [141.36–212.04]	
Cost per person where eradication failed (GP visit, treatment, complications)	$129.85 [103.88–155.82]	

Confidence intervals (CI) (95%) and uncertainty ranges (also assumed to be 95%) were used to calculate standard deviations for uncertainty intervals (UIs) using a Beta distribution for proportions and a normal distribution for scalars. There are also multiple baseline input parameters not included in this table (e.g. gastric cancer rates by sex by age by ethnic group, competing background mortality rates and health system costs for a gastric cancer patient); see text

*OAC* omeprazole, amoxicillin and clarithromycin, *OBTM* omeprazole, bismuth/De-Nol, tetracycline and metronidazole, non-Māori includes Pacific, Asian, European and Other ethnic groups

^a^Contributes to the effect size and the screening pathway

^b^Applied the same standard error as for the OAC eradication rate

1. The proportion of gastric cancer that was non-cardia was set to the New Zealand distribution of non-cardia and cardia gastric cancer, excluding overlapping and undefined subtypes (2007-2011 in New Zealand, Cancer Trends). It varied by ethnicity and sex but was generally stable by age.
2. We set 89% as the proportion of non-cardia gastric cancer attributable to *H. pylori* (population attributable fraction, PAF) [1], based on three prospective studies from Europe and Australia, where *H. pylori* tested by immunoblot was present in nearly 95% of non-cardia gastric cancer cases [1]. It was not possible to allow the PAF to vary by time and subgroup.
3. Coverage of screening in the main model was set according to the coverage of cardiovascular risk assessments (CVRA) for adults in New Zealand [27] adjusted for primary care enrolment, namely 81% for Māori and 84% for non-Māori. A scenario analysis including ‘equal coverage’ by ethnicity was also run. Both CVRA and *H. pylori* screening are administered though primary care and involve blood tests in adults. CVRAs are a funded government target.
4. The sensitivity of screening was set at 89% for serology (and 95% for fecal antigen in a probabilistic scenario analysis) in line with the literature [5, 28].

The effect size was modeled to respond to different eradication rates by ethnicity, at least partially due to differences in *H. pylori* clarithromycin resistance [29]. This was set using the relative eradication rate in New Zealand [29] compared to a pooled eradication rate from the meta-analysis (with the addition of 60% eradication for people who failed first line therapy and were retreated [16]).

In developing the model we assumed that the relative risk reduction of gastric cancer in *H. pylori* positive participants (largely dependent on one large trial in China) [2] would be similar in high and low gastric cancer incidence countries. The effect size was estimated to apply over a lifetime of follow-up, similar to other cost-utility analyses (CUAs; Table 3), and the reinfection rate in screened adults was assumed to be 0% given transmission predominantly occurs in infants and children [5]. A CUA in the UK that included a reinfection rate of 0.3% in a scenario analysis, found it had a small impact on cost-effectiveness [13].

It is plausible that the effect of treatment may be greater in younger people because they have no pre-existing metaplastic changes. However, a significant effect size was evident in 55+ year olds in China [30] and there was no statistical evidence from a meta-analysis that the effect differed by presence of pre-neoplastic lesions [2]. We therefore included all ages in this analysis, assumed the effect size to be the same across age groups, and suggest future trials and meta-analyses stratify results by age wherever possible.

#### Costs

The downstream healthcare system costs and benefits that were incurred or averted (offset) as a result of the intervention were calculated from the health sector perspective. Costs and benefits outside the health system such as benefits to worker productivity were therefore out of scope. Costs were reported in 2011 New Zealand dollars (NZ$) and exclude a value-added tax (GST). Selected results are presented in United States dollars (US$) using the OECD 2011 purchasing power parity (2011: 1 NZ$ = 0.67 US$) [31]. A 3% discount rate was applied to both costs and benefits. Costs of the screening program were obtained from current health system settings and adjusted to 2011 NZ$ using the New Zealand consumer price index [32].

The cost of publicly-funded health services by age was from the New Zealand Ministry of Health database, called ‘HealthTracker’. HealthTracker is a collection of linked administrative datasets of publicly funded health system events (including hospitalizations, mortality, cancer registrations, mental health and addiction service use, pharmaceutical and laboratory claims, primary health care enrolment, and outpatient/emergency department visits) for the entire New Zealand population with unit costs attached [33].

In the ‘healthy’ and ‘cancer’ states individuals were assigned average population health system costs and average population morbidities. Additionally, in the “gastric cancer” state individuals were assigned the excess costs of gastric cancer. The average health systems costs in the last year of life were assigned to those who died from other causes and the excess cost of gastric cancer in the last year of life was assigned to those who died from gastric cancer. More information on costing is available in the Additional file 1 and in study costing protocols [34, 35].

#### Screening program costs

For each strata of age, sex and ethnicity, the cost per person screened was assigned based on fixed costs applied to the total population invited to be screened, costs per person tested, costs per expected rate of positive serology, and finally costs per positive retest (see Additional file 1). Costs included patient co-payments for primary care and pharmaceuticals.

Perhaps the most well-defined adverse effect of triple therapy is the risk of *Clostridium difficile* infection (CDI) with severe diarrhea. The cost of CDI was estimated as a 1–12 day hospital admission with severe diarrhea (using cost weights for hospital events). We set the expected rate of CDI at 80 per 100,000 people treated, according to an estimated 11 per 100,000 incidence of CDI in the community, and 2.65 times greater risk of CDI with macrolide treatment and 2.71 times greater risk with penicillin [36]. The disability weight was not included in the model because it was too small to affect QALYs^DW^ gained given that CDI is rare.

### Analyses

#### Uncertainty analysis

Monte Carlo simulation techniques were used for probabilistic sensitivity analysis to test the uncertainty in the model outputs. This involved selecting key input parameters probabilistically from their distributions using Excel and Ersatz software (Table 1) with 2000 simulations.

#### Scenario analyses

Cost-effectiveness in the total population, and for Māori and non-Māori subgroups, was examined deterministically for several scenarios including discounting, equity analyses, low coverage, 15 years of follow-up, use of levofloxacin for greater eradication success, and removing the retest step.

## Results

### Main analysis

The *H. pylori* screening program cost was estimated at NZ$24,600 ($11,300-$57,400) (US$16,500) per QALY gained for the total population. The targeted screening program for Māori was more cost-effective at $11,985 ($5,719-$27,564) (US$8,030) per QALY gained.

Compared to current practice, the total net cost of a one-off total population *H. pylori* screening program for 25-69 year olds (with lifetime follow-up for downstream health system costs) was $293 million (95% uncertainty interval [UI]: $272-$314, probabilistic model, discount rate 3%) (US$196 million) (Table 2). The net cost was made up of $294 million ($282-$307) in intervention costs, and $1.5 million (-$23-$26) due to downstream future health system cost savings from reduced gastric cancer incidence and associated treatment costs. In comparison, a targeted *H. pylori* screening program (for Māori only) had a net cost of a $41 million ($35-$46) (US$27 million).

**Table 2:** Incremental costs, QALYs gained and ICERs for the total New Zealand adult population including a subset of the population with high risk of gastric cancer (Māori), comparing serology and fecal antigen as the screening test, in 2011 with lifetime follow-up of participants

Model output	Total population 25–69 yo	Māori 25-69 yo	Non-Māori 25–69 yo
Serology based screening			
Men and women			
Number cases of gastric cancer averted	3658 (1252–4425)	1007 (342–1828)	2650 (905–4837)
Percentage cases of gastric cancer averted	16.5% (5.6%–29.4%)	21.6% (7.4%–38.6%)	15.2% (5.2%–27%)
Number of gastric cancer deaths averted	2434 (834–4425)	714 (242–1293)	1720 (588–3141)
Percentage gastric cancer deaths averted	16.6% (5.7%–29.5%)	21.6% (7.3%–38.6%)	15.2% (5.2%–27%)
Total net incremental cost (NZ$ million)	$293 ($272–$314)	$41 ($35–$46)	$252 ($233–$272)
Total intervention cost (NZ$ million)	$294 ($282–$307)	$41 ($38–$45)	$253 ($242-$264)
Total cost offsets (NZ$ million)	-$1.5 (-$26.2–$22.9)	-$0.61 (-$7.35–$6.21)	-$0.89 ($-22.7–$21.7)
Total QALYs gained	14,200 (5100–26,300)	4000 (1400–7400)	10,200 (3653–18974)
Incremental net cost per participant (NZ$)	$119 ($111–$128)	$137 ($117–$158)	$117 ($108–$126)
Incremental QALYs gained per participant	0.0058 (0.0020–0.0107)	0.0137 (0.0047–0.0252)	0.0047 (0.0016–0.0087)
ICER (NZ$ per QALY gained)	$24,600 ($11,300–$57,400)	$12,000 ($5700–$27,600)	$29,600 ($13,400–$69,800)
Men			
Incremental cost per participant (NZ$)	$123 ($113–$134)	$147 ($123–$173)	$120 ($110–$131)
Incremental QALYs gained per participant	0.0071 (0.0024–0.0131)	0.0158 (0.0055–0.0293)	0.0059 (0.0020–0.0110)
ICER (NZ$ per QALY gained)	$20,800 ($9800–$47,900)	$11,000 ($5600–$24,300)	$24,300 ($11,300–$57,100)
Women			
Incremental cost per participant (NZ$)	$116 ($108–$124)	$129 ($110–$148)	$114 ($105–$123)
Incremental QALYs gained per participant	0.0046 (0.0016–0.0085)	0.0118 (0.0040–0.0217)	0.0036 (0.0012–0.0067)
ICER (NZ$ per QALY gained)	$30,200 ($13, 400–$71,400)	$13,200 ($5900–$31,300)	$38,000 ($16,800–$89,900)
Fecal antigen based screening			
Men and women			
Total incremental cost (NZ$ million)	$369 ($350–$389)	$49 ($44–$55)	$320 ($301–$339)
Total QALYs gained	15,300 (5400–27,700)	4200 (1500–7600)	11,000 (3830–20,200)
Incremental cost per participant (NZ$)	$150 ($142–$158)	$164 ($147–$182)	$148 ($139–$156)
Incremental QALYs gained per participant	0.0061 (0.0022–0.0111)	0.0142 (0.0051–0.0259)	0.0050 (0.0018–0.0092)
ICER (NZ$ per QALY)	$29,000 ($13,600–$65,900)	$13,700 ($6700–$30,500)	$34,900 ($16,300–$79,100)

Central estimates are the mean from the probabilistic sensitivity analysis Monte Carlo simulations and the brackets indicate the 95% uncertainty intervals

*QALY* quality-adjusted life year with disability weights, *ICER* incremental cost-effectiveness ratio

The health gain from the population screening program was estimated at 14,200 QALYs (95% UI: 5100-26,300) over the cohort’s lifetime. This corresponds to 3660 (1250–4430) fewer cases of gastric cancer, 2430 (830–4430) averted deaths from gastric cancer, and a 17% (6%–29%) reduction in the expected future gastric cancer cases and deaths. The health gains for Māori were greater per person with a 22% (7%–39%) reduction in the expected Māori gastric cancer incidence and mortality.

For non-Māori, the cost per QALY was over twice that for Māori at $29,600 per QALY gained ($13,400–$69,800) (US$19,900). Within the non-Māori population there was marked ethnic heterogeneity, with 15% who were Asian and 7% who were Pacific (2013 census, 25–69 years old). Pacific people have greater gastric cancer rates than European Other peoples.

**Fig. 2:**
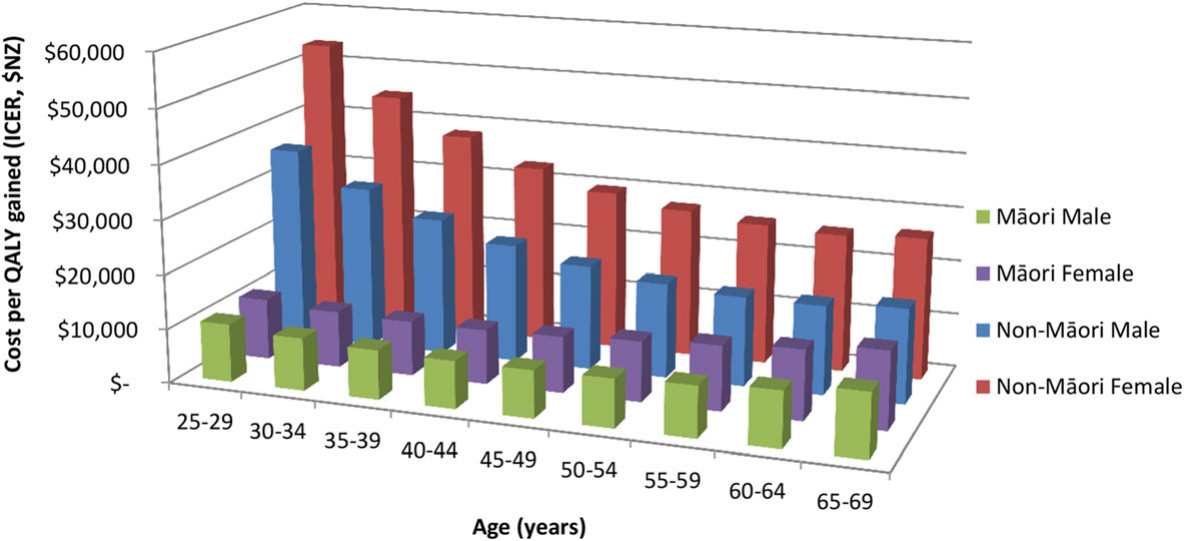
Modeled cost-effectiveness of a *H. pylori* screening program in New Zealand by ethnicity, sex and age for the 25-69 year old population in 2011, expected value (deterministic analysis), NZ$ 2011. *See supporting documents for values on this graph

Variation in cost-effectiveness by age was explored for Māori and non-Māori subgroups (Fig. 2). Cost-effectiveness for Māori was greatest in the 45–49 year old age group and for non-Māori in the 60–64 age group, likely due to relatively higher Māori rates of gastric cancer at younger ages.

**Fig. 3:**
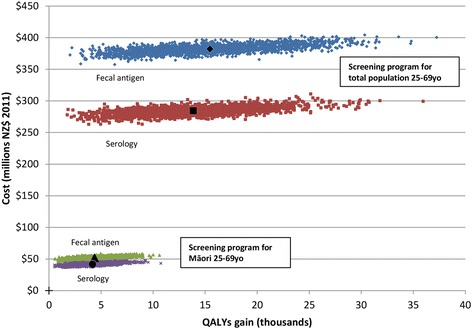
Incremental costs and QALYs gained from a *H. pylori* screening program for Māori and the total population, comparing serology versus fecal antigen screening tests

Compared to current practice, the fecal antigen test was less cost-effective (ICER of $29,000 UI: $13,600-$69,200 or $US 19,400) than serology-based screening ($24,600) (Table 2, Fig. 3). More specifically, the potential QALYs gained with fecal antigen testing were 7.5% greater (15,300 vs 14,200 QALYs) but the cost was 26% greater ($369 million vs $293 million). The incremental cost-effectiveness of fecal antigen compared to serology testing was $71,700 per QALY gained.

### Uncertainty analysis

Probabilistic sensitivity analysis demonstrated that at a willingness-to-pay of NZ$45,000 per QALY, there was greater than 99% probability that a serology-based screening program for Māori men and women would be cost-effective. For non-Māori men and women the probability was 94% and 77% respectively. The greatest contributor to uncertainty in the model was the effect size of *H. pylori* eradication (RR 0.66, 95% CI: 0.46-0.95) [2]. At the extremes of the likely effect size, the cost-effectiveness of a screening program for the total population was between $12,000 and $58,200 per QALY gained.

### Scenario analyses

Given the higher background mortality and morbidity for Māori, a gastric cancer death prevented among Māori typically achieves less health gain than a gastric cancer death prevented among non-Māori (Table 3). Therefore, we ran an ‘equity analysis’ where the Māori analyses were rerun with non-Māori background mortality and morbidity rates. This resulted in a 36% increase in QALYs (Table 3). In an equal coverage scenario Māori analyses were rerun with non-Māori coverage, resulting in 4% greater QALY gains for Māori. When analyses were rerun with low screening coverage adopting participation rates from the first year of a colorectal screening pilot program in one New Zealand health district (Waitemata), the QALY gains were 40% less for Māori and 30% less for total population.

**Table 3:** Scenario analyses and their impact on health gain, health system costs, and incremental cost-effectiveness ratios per person screened, New Zealand dollars 2011

	Incremental costs (NZ$)			Incremental QALYs gained			Incremental cost-effectiveness ratio		
	Total	Māori	Non-Māori	Total	Māori	Non-Māori	Total	Māori	Non-Māori
Main model^a^	$119	$138	$117	0.006	0.014	0.005	$20,600	$10,100	$24,700
Equity analysis—equal life expectancy and background morbidity for Māori and non-Māori	$121	$155	$117	0.006	0.019	0.005	$18,800	$8,200	$24,700
Equal coverage—coverage in Māori was set to the same as non-Māori (84%)	$120	$143	$117	0.006	0.014	0.005	$20,400	$10,000	$24,700
Low coverage—coverage in Māori of 45% and non-Māori of 58% akin to a NZ colorectal screening pilot	$93	$95	$93	0.004	0.008	0.003	$24,500	$12,400	$28,400
Equal eradication—triple therapy was increased to be 95% effective (e.g. levofloxacin)	$118	$132	$116	0.006	0.015	0.005	$19,600	$8,900	$24,200
No retest to ensure effective eradication	$101	$107	$100	0.005	0.010	0.004	$20,600	$10,900	$23,600
Follow-up for 15 years (rather than over a lifetime)	$118	$132	$116	0.003	0.008	0.003	$35,600	$16,000	$44,000
The effect size in the youngest age groups is greater (<40yo, RR of 0.50)	$119	$139	$117	0.006	0.016	0.005	$18,400	$8,700	$22,500
The complication rate for CDI was increased from 80 to 800 per 100,000	$124	$146	$121	0.006	0.014	0.005	$21,400	$10,700	$25,600
6% discounting QALYs and costs	$112	$118	$111	0.003	0.007	0.002	$39,200	$17,300	$48,200
0% discounting QALYs and costs	$151	$225	$141	0.015	0.034	0.012	$10,300	$6,600	$11,700
No unrelated health system costs	$82	$52	$86	0.006	0.014	0.005	$14,200	$3,800	$18,300
No pYLDs (background morbidity)	$119	$138	$117	0.008	0.021	0.007	$14,300	$6,700	$17,500

Deterministic results were used in the sensitivity analysis for efficiency and differ slightly from probabilistic results

^a^Main model includes 3% discounting of both QALYs and costs

The screening program was considerably less cost-effective when the treatment effect was applied for a limited follow-up period of 15 years (ICER was 73% greater; the maximum follow-up in the most relevant meta-analysis was 15 years). Discounting (costs and benefits) had a large impact on cost-effectiveness (particularly on QALYs) reducing the ICER by 50% with no discounting; and increasing the ICER by 90% for 6% discounting. Substituting a lower screening coverage in the model (45% for Māori and 58% for non-Māori) caused the ICER to increase by 19% (Table 3).

## Discussion

### Main findings and interpretation

In this analysis from a high income country, the cost-effectiveness of targeted screening in a high gastric cancer incidence group (Māori ICER $12,000, UI $5,700–$27,600) was more favorable than for the total population (ICER $24,600, UI $11,300–$57,400). These findings are analogous to US findings where cost-effectiveness of screening was better for Japanese American and African American participants [8, 17].

Our results for the non-Māori group (ICER of NZ$24,700, expected deterministic result, Table 3) include populations with low and high gastric cancer rates (such as Pacific peoples). Whilst we did not have data for a full ethnicity-disaggregated model, if we assume that the intervention costs are distributed pro-rata by population counts, and that the health gain is distributed proportional to gastric cancer incidence (2007–2011), then we roughly estimate that the cost-effectiveness would be $8,300 for Pacific (76% less than non-Māori altogether), $29,000 for Asian and $28,800 for European/Other (both the later were 17% greater than non-Māori aggregated). These estimates are very approximate however and do not allow for different age structures, different background mortality and morbidity rates, and heterogeneity within the Asian group. At this level of cost-effectiveness for European/Other the uncertainty in treatment effect size and differences in cost-effectiveness by age and sex become particularly relevant to decision makers. We conclude that *H. pylori* screening is probably of borderline cost effectiveness for European/Other, but probably cost-effective for Pacific people as well as Māori.

Variation in results by age and sex reflect the epidemiology of non-cardia gastric cancer. The intervention was particularly more cost-effective in younger Māori adults (45–49 year olds). Among Māori cost-effectiveness was similar in men and women, consistent with findings from a high risk region of China [19]. In non-Māori, cost-effectiveness peaked in participants aged 60–64 years old similar to studies in the UK [13] and US [7]; and *H. pylori* screening was more cost-effective in men, similar to US findings [17].

Serology based screening was more cost-effective than fecal antigen based screening (Fig. 3). The incremental cost effectiveness of moving from serology to the fecal antigen test was $71,700 per QALY gained. Although the fecal antigen test has a preferable sensitivity (95% vs 89%) and is likely to result in greater health gains, the cost of the test was problematic (fecal antigen test cost was $65.20 vs $30.68 for serology). Reduction in the cost of fecal antigen testing would shift the balance towards making fecal antigen based screening more cost-effective than serology. The fecal antigen test is widely used in many high-income countries, and there may be potential to introduce it in combination within a country’s national colorectal screening program (if differences in stool sample requirements can be addressed). Fecal antigen may however adversely impact on screening uptake given likely participant dislike of dealing with fecal material. Conversely, *H. pylori* serology could be added onto existing blood test requests in primary care as part of the routine screening for other conditions (such as diabetes and cardiovascular risk in the New Zealand case).

### Strengths and limitations

The key strengths of this CUA are the adoption of a recent meta-analysis effect size for *H. pylori* eradication [2], presenting incremental costs per QALY gained, using probabilistic sensitivity analysis, and allowing for adverse effects of treatment costs (CDI). Our input variables included high quality New Zealand ethnicity, cancer registration and cost data. Meta-analysis results from high risk populations [2] were shaped to fit the New Zealand context by taking into account differences in non-cardia gastric cancer incidence, the PAF for *H. pylori* and gastric cancer [1], New Zealand eradication rates for *H. pylori* treatment [29], and the declining incidence of gastric cancer. We compared results by age, sex and ethnicity including for a population with relatively high rates of gastric cancer (Māori).

Nevertheless, the modeled benefits of our screening program are likely to be underestimated as we did not have the primary care data to include the health gains and cost reduction benefits from reduced dyspepsia and peptic ulcers attributable to *H. pylori* infection in this context. However, screening studies in the UK have demonstrated a 25% reduction in dyspepsia after two years of follow-up, with a mean saving of dyspepsia-related costs of US$117 per person ($11–$220) in the 10 years after eradication therapy [37]. If this cost-saving from dyspepsia was the same in the New Zealand context, cost-effectiveness may be greater in our model. For example, if we used UK dyspepsia-related cost-savings, and subtracted these from the model’s costs in 45-49 year olds seropositive for *H. pylori* over a ten year time line, this would reduce the ICER by an estimated 24% in Māori and 20% in European/Other, even when not considering the likely QALY gain from dyspepsia relief (see Additional file 1: Table S8 for this calculation). Furthermore, other than CDI, we were unable to account for potential harms from *H. pylori* eradication treatment however adverse effects are often rare and inclusion is unlikely to influence our findings. Similarly, we did not account for any increased anxiety among those screened and told that they have *H. pylori* infection (or the reassurance value when told that they are free of infection).

This CUA was based on *H. pylori* prevalence and gastric cancer incidence in New Zealand for 2011 in all ages from 25 to 69 years. Cost-effectiveness may gradually decrease over time as *H. pylori* prevalence and gastric cancer incidence decline. It will take several decades however for the current high risk birth cohorts to be replaced by younger cohorts at low risk of *H. pylori* infection. *H. pylori* screening in populations with high gastric cancer incidence such as Māori appears likely to remain cost-effective for some decades into the future. Cost-effectiveness also depends on which age group is screened.

Furthermore, positive serology does not represent an active infection and some participants are likely to be treated without having *H. pylori* in their stomach. This could be remedied somewhat by using a fecal antigen test instead (as modelled) or the more expensive options of a urea breath test or a two-way diagnostic procedure. The latter would have a greater chance of losing people over two steps. Also if participants are not tested after a second therapeutic attempt a small proportion (3–4%) may still have *H. pylori* infection. To avoid falsely reassuring treated individuals, information materials should state that treatment is not 100% effective and if they get gastric symptoms in the future they should consult their doctor.

### Practice, policy and research

The WHO recommends that countries should consider piloting *H. pylori* screening to evaluate its impact on gastric cancer, all-cause mortality and potential adverse effects [5]. Given current evidence, our results indicate that screening and treating definable ethnic groups with a high risk of developing gastric cancer is likely to be the most cost-effective approach in high-income country settings. Screening may also be cost-effective in populations with low incidence of gastric cancer but more evidence is probably needed to establish more precise estimates of screening benefits and costs by age, sex and risk status.

There are many other factors that should be considered before the introduction of screening programs [38]. More information is required about the balance of harms and benefits of screening, the acceptability and feasibility of *H. pylori* infection screening tests, the capacity of the health care system particularly primary care, and potential ethical issues about identifying infection and being unable to treat it in some cases. Policy-makers would also need to consider the relative cost-effectiveness and potential equity gains of alternative interventions in high-income countries such as tobacco control, obesity control and other screening programs (for example colorectal cancer, chronic kidney disease and lung cancer in ex-smokers).

Trials of population based *H. pylori* screening in participants as young as 25 years and up to 69 years, are under way in Linqu Country, China; United Kingdom; Matsu Islands in Taiwan; Korea and another is planned for Latvia, Belarus and the Russian Federation [39]. Emerging evidence should be actively monitored to clarify the potential adverse effects of *H. pylori* screening and treatment (such as all-cause mortality outcomes from screening trials), and the most effective models of implementation. Potential adverse effects from *H. pylori* treatment require further evaluation since confounding is a likely contributor to inverse associations demonstrated between *H. pylori* infection and esophageal cancer, asthma, ischemic heart disease, colon cancer and antibiotic resistance [40]. Nevertheless, given what is known to date it would seem reasonable for policy-makers in high-income countries to start exploring the option of pilot screening programs for *H. pylori*, especially in population groups with relatively high rates of gastric cancer.

## Conclusions

This CUA modeling study found that a *H. pylori* screening program was cost-effective in this high-income country setting for the indigenous Māori population (and likely by extension to Pacific peoples). The program was of likely borderline cost-effectiveness for the European/Other population. A screening program may therefore help to address the existing inequalities in the burden of gastric cancer. While further research will help clarify the precise benefits, costs and adverse effects of such screening programs, there seems a reasonable case for policy-makers to give consideration to establishing pilot programs now, particularly for any population groups with relatively elevated rates of gastric cancer.

## Additional file

Additional file 1: Supplemental Material: Further information on the methods and inputs. (DOCX 122 kb)

## Abbreviations

CDI: *Clostridium difficile* infection
CUA: Cost-utility analysis
CVRA: Cardiovascular Risk Assessment
GST: Goods and services tax (a value added tax)
*H. pylori*: *Helicobacter pylori*
ICER: Incremental cost-effectiveness ratio
OECD: Organisation for economic co-operation and development
QALY^DW^/QALY: Modified quality-adjusted life-year
RR: Rate ratio
WHO: World Health Organization
