# Supplementary material for: A screening program to test and treat for Helicobacter pylori infection: Cost-utility analysis by age, sex and ethnicity
Source: BMC Infect Dis. 2017 Feb 20;17:156. doi: 10.1186/s12879-017-2259-2 (PMC5319166; doi:10.1186/s12879-017-2259-2)
Supplement: Additional file 1: — Supplemental Material: Further information on the methods and inputs. (DOCX 122 kb) [file 12879_2017_2259_MOESM1_ESM.docx]

# Additional file 1

Further information on the methods and inputs

## Further introduction

### Cost-effectiveness analyses

Table S1: Examples from previous cost-effectiveness analyses of *H. pylori* population screening programs. Adapted from I Lansdorp-Vogelaar and L Sharp [1] and the International Agency for Research on Cancer and World Health Organization [2]

| Country &  Study | Heterogeneity |  | Methods |  |  |  |  | Results |
| --- | --- | --- | --- | --- | --- | --- | --- | --- |
|  | *Population*  *(*gastric *cancer incidence per 100,000 population)* | *Age (years)*  *(sensitivity analyses)* | *Uncertainty analysis* | *H. pylori test* | *Screening program scenarios* | *H. pylori treatment effect size* | *Type of analysis* | *ICER* |
| USA[3] | Sexes combined:  African American (12.6)  Japanese American (28.5)  White (6.8) | 50-54 | One-way sensitivity analysis | Serology | - Screen & treat  - Treat everyone | 0.70 (0.30- 0.95)  distal cancer | C/LYS | US$ 25,000  CU was best for Japanese Americans |
| USA[4] | Sexes combined:  USA (10)  Colombia (44.3)  Finland (21.8)  Japan (110.6) | 50-54  (10, 20, 30, 40, 60 & 70) | One-way sensitivity analysis | Serology | - Screen for all *H. pylori*  - Screen for Cag A | 0.70 (0.30-0.95)  distal cancer | C/LYS | US$ 23,900  CU was best in high incidence countries |
| USA[5] | White men & women separately  African American men  Hispanic men  Japanese men | 40 | One-way sensitivity analysis | Serology | -screen & treat, with retest  - screen & treat, without retest | 0.28 (0-0.5)  (RR 3.6)  gastric cancer | C/LYS | US$ 6,264 |
| England[6] | Sexes combined:  General population | 40-49 | One- and two-way sensitivity analysis | Serology | - screen and treat | 0.30 (0.12-0.60)  distal  cancer | C/LYS | Saves US$ 9 per person screened &  130 LYS per 105 people screened |
| England & Wales[7] | Sexes combined:  General population  (1960-69 birth cohort used for incidence of gastric cancer) | 40-49  (20-49)  (30-49)  (50) | Probabilistic | Serology,  UBT | - screen and treat (assuming a degree of opportunistic screening) | 0.33 (0.13-0.50)  (RR 3, but used age specific risks)  gastric cancer | C/LYS | US$ 8,800 (at age 40) |
| Taiwan[8] | Sexes combined:  General population Matsu Islands, Taiwan | 30  (50) | Probabilistic | UBT | - screen & treat once only  - annual screen & treat | 0.64  (RR 1.6)  gastric cancer | C/LYS | US$ 17,044 at age 30 |
| Singapore[9]^,^ [10] | General population Chinese men | 35-44 | One-way sensitivity analysis | Serology,  UBT | - screen and treat | 0.70 (0-1)  gastric cancer | C/QALY | US$ 13,571 |
| China[11] | General population Linqu, China (high risk area)  Men & women separately | 20, 30, 40, 50, 60 | One-way sensitivity analysis | Serology | - screen and treat once only  - screen and treat, rescreen if negative results | 0.85 (0.70-0.93) in 20yo, 70% Hp+ve:  varies by sex and natural history parameters | C/LYS | < US$ 1,600 per LYS for each age group |
| Canada[12] | General population  Men (6.6) | 35 | Probabilistic | Serology,  UBT , Stool antigen test | - screen and treat | 0.70 (0.69-0.71)  (RR 1.42)  gastric cancer | C/QALY | US$ 33,000  (stool antigen test most cost-effective) |
| Taiwan[13] | Male and female | 30-39,  40-49,  50-59,  60-69,  70-79, 80+ | One- and two-way sensitivity analysis | UBT, serology | - screen and treat | 0.63  gastric adenocarcinoma | C/LYL | Females US$244/LY, men $312/LY |

C, cost; DES, discrete event simulation; LYS, life-year saved; QALY, quality-adjusted life-year; LYL, life-year lost

## Further methods

### Screening pathway

Figure 4: *H. pylori* screening program pathway used in the main model


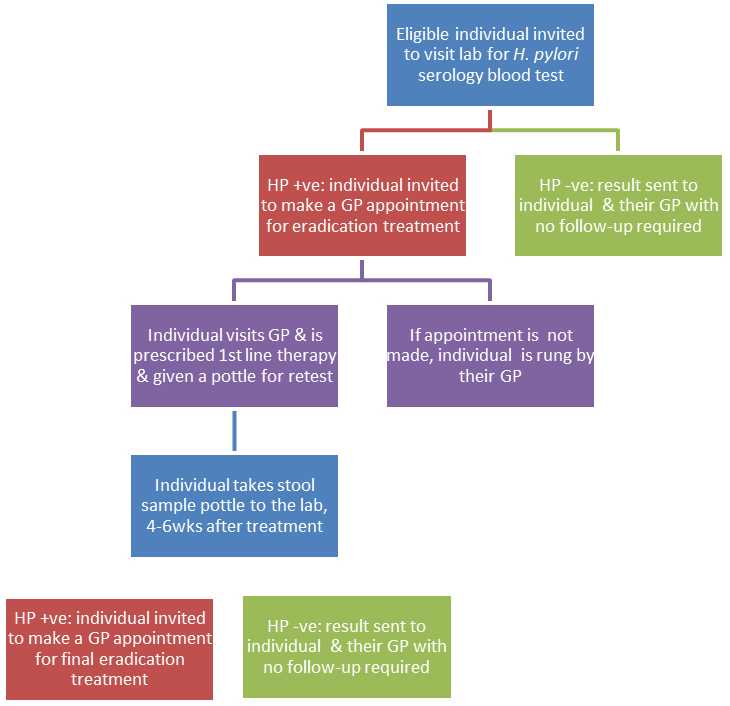


### The effect size equation

$${Incidence of preventable gastric cancer \left( age, sex, eth \right)}={Incidence}_{gastric cancer}\times{Prop}_{non-cardia}\times PAF\times Coverage \times{Sensitivity}_{sero}$$

$$Effect size\left( age, sex, eth \right)=1-\left[ \left\{ 1-{RR}_{Ford 2014} \right\}\times\frac{{ER}_{NZ}}{{ER}_{Ford 2014}} \right]$$

### Cost of screening for *H. pylori* using a serology test

These domains were used to cost the screening program;

1. Cost per invitation – fixed costs per person applied to all of the population invited for screening. Costs included public awareness, material development, screening register, District Health Board (DHB) and screening unit overheads and the coordination center. We excluded Ministry of Health development costs and oversight costs for ongoing monitoring, governance and policy, given that salary costs are not likely to be any extra to current costs.
2. Cost per uptake of testing – applied to the expected coverage; estimated using New Zealand cardiovascular risk assessment coverage for Māori (81%) and non-Māori (84%), adjusting for population size and PHO enrolment rates.[14] Costs included the serology test, courier, consent forms and results letter.
3. Cost per positive *H. pylori* result – applied to the expected *H. pylori* seroprevalence; estimated using pooled seroprevalence from New Zealand studies by ethnicity and cohort of birth.[15] Costs included a general practitioner appointment, pharmaceuticals, courier, fecal antigen test, results and CDI.
4. Cost per failed eradication – applied to the complement of the expected ethnicity-specific eradication rate; estimated from a recent New Zealand study(South Auckland) that used an intention-to-treat analysis.[16] Costs included a general practitioner appointment, pharmaceuticals and CDI.

Table S2 : Breakdown of screening program costs per person by domain – the total cost per person depends on weighting given to each domain

| Domain | Variable | Cost  (NZ$ 2011) | Reference | Assumptions |
| --- | --- | --- | --- | --- |
| Costs per person invited  (fixed costs) | Fixed per person costs of screening program including community awareness raising, material development, coordination center, DHB overheads, pilot register, screening unit fixed costs ($2.651m, 2013, across 72,484 people invited) | 35.82 | Colorectal screening pilot | Assuming colorectal screening program costs are similar to *H. pylori*. This may be over estimate because endoscopy unit costs are included but not relevant. |
|  | Cost of pre-invitation letter, brochures, invitation postage, and reminder letter at four weeks for 60% people | 4.05 | Colorectal screening pilot | Colorectal screening program costs are likely to be similar to *H. pylori* screening. |
| Costs per person tested  (variable cost) | Return consent forms to the Coordination Center, results letter and brochure sent to individual, any postage or courier costs | 23.98 | Colorectal screening pilot | Assuming colorectal screening program costs are about double *H. pylori* costs because the latter won't require postage to the lab |
|  | Cost of lab test | 30.68 | See below | *H. pylori* serology blood test cost is the average of three laboratory quotes adjusted to 2011 cost using CPI. |
| Costs per person with a positive test  (variable cost) | Standard GP appointment cost (government subsidy + average co-payment) | 63.46 | BODE^3^ protocol [17] | Assuming all who test positive see a GP. |
|  | Total acquisition cost of triple therapy, including government subsidy, pharmacy margin, pharmacy service fee and patient co-payment | 17.85 | [18] | OAC: omeprazole 20mg ($2.91*14/90), amoxicillin 1g ($20.94*28/300) (*Substitute with metronidazole for +$0.59) and clarithromycin 500mg ($10.40) twice daily for 7 days |
|  | Cost of retest kit consumables (pottle and collection sheet) | 3.13 | Colorectal screening pilot | Colorectal screening test kits are similar to *H. pylori* fecal antigen test. |
|  | Return consent forms to Coordination Center, results letter and brochure sent to individual, any postage or courier costs | 23.98 | Colorectal screening pilot | Assuming colorectal screening program costs are about double *H. pylori* costs because the latter won't require postage to the lab. |
|  | Cost of fecal antigen lab test to test to investigate if there has been effective eradication | 65.20 | See below | Fecal antigen test cost is the average of three laboratory quotes adjusted to 2011 using CPI. |
|  | Average cost of complications (risk *Clostridium difficile* infection X hospitalization cost) | 3.09 | BODE^3^ protocol [17] WIES14 | All cases of antibiotic related CDI are hospitalized. |
| Costs per person where triple therapy eradication failed (variable cost) | Standard GP appointment cost (government subsidy + average co-payment) | 63.46 | BODE^3^ protocol [17] | All who test positive are treated. |
|  | Total acquisition cost of quadruple therapy, including government subsidy, pharmacy margin, pharmacy service fee and patient co-payment | 63.30 | BODE^3^ protocols [17-19] | Assume all people with failed eradication are retreated with:  omeprazole 20mg bd (2.91*14/90), De-Nol/ tripotassium dicitratobismuthate tabs 2 bd (28/112*32.50), tetracycline HCl 500mg qid (46.00), and metronidazole 400mg tds (18.15/100*21) for 7 days. |
|  | Average cost of complications (as above) | 3.09 | BODE^3^ protocol [17] WIES14 | Assume same rate of complications for triple therapy and second line quadruple therapy. |

#### Pharmaceuticals

Recommendations regarding treatment are outlined in the screening pathway section.

Total payment for each pharmaceutical (excluding GST) was calculated according to the BODE^3^ protocol [19] and includes:

- GST-exclusive subsidy (Sc) as listed in the Pharmaceutical Schedule plus
- the pharmacy margin (M) on the subsidy (Sc) plus
- the base pharmacy services fee (BPSF) adjusted by the appropriate multiplier (F)(F=1, BPSF=$5.30)

Total government cost = [Sc + (Sc*M) + (BPSF*F)]

Levofloxacin 500mg daily has been suggested to Pharmac but has not been made available due to difficulty finding a company to supply it to New Zealand. The costs online tend to be similar to the clarithromycin that it would replace e.g. OAL rather than OAC as first-line therapy. Levofloxacin was run as a scenario analysis with a proposed eradication rate of 90% for all groups.

#### Test costs for H. pylori screening

The following table refers to the laboratory test costs for each of the tests.

Table S3: Test costs for *H. pylori* screening

| Costs (New Zealand, 2015) | *H. pylori* fecal antigen | *H. pylori* Serology IgG |
| --- | --- | --- |
| Canterbury Health Laboratories http://www.labnet.health.nz/testmanager/index.php?fuseaction=main.displaytest&testid=1018 | Cost $73.69 excl GST | $30.78(Exclusive of GST) |
| Auckland DHB Lab Plus  http://testguide.adhb.govt.nz/EGuide/ | External price incl GST is $60.42 (excl. GST $53.71) |  |
| Waikato DHB Laboratory Website  http://lab.waikatodhb.health.nz/test-guide/view/400/h-pylori-igg |  | External Price (excl. GST) $26.43 |
| Laboratory tests http://www.labtests.co.nz/patients/general-information/test-pricing-correct | $85.00 incl GST (excl. GST $75.56)  (cost to public marked up) | $43.00 incl GST (excl. GST $37.39)  (cost to public marked up) |
| Average cost: | **$67.65**  (rounded to 68, uncertainty 50-80) | **$31.53**  (rounded to 32, uncertainty 20-40) |

#### Comparison of laboratory tests for H. pylori

Serology was selected as the base case after consideration of the factors below. Fecal antigen was run as an alternative scenario by adjusting the screening program costs, improved effectiveness due to greater test sensitivity and reduced cost due to a reduction in false positives.

Table S4: Logistics, feasibility and acceptability of two tests for *H. pylori*

| *H. pylori* Serology IgG | *Helicobacter pylori* fecal antigen |
| --- | --- |
| Not listed on the schedule for funding | Funded, Terre 1 in Laboratory Schedule |
| Do not need to stop proton pump inhibitor medications or antibiotics.  Inferior sensitivity and specificity as to diagnose *H. pylori* infection. | Must stop omeprazole, bismuth compounds and antibiotics for 2 weeks before having the test. |
| Will require phlebotomy. A patient normally attends the lab for a community blood test. The laboratory phlebotomist is covered in bulk funded contract, but a screening program may require additional compensation.  On occasions a GP may take the blood at the surgery to ensure the test is done. | GP normally gives a patient a container, collection pottle and lab form / biohazard bag. The patient then needs to drop sample into the lab on the same day. Bacterial over growth can make the test less sensitive (rarely occurs). Some labs say sample must be refrigerated <24hrs, frozen if >24hrs. Others say must arrive within 48 hours between collection and arrival at the lab. (Colorectal sample can be at ambient temperature). Rarely does the lab identify any problems with invalid samples because of overgrowth. |
| Blood tests are routinely done and generally considered more acceptable. | Patients often dislike this test. However it is also non-invasive. |

### Scenario analysis methods

Table S5: Equity analysis methods

| Name | Equity analyses |
| --- | --- |
| Standard analyses | 1. Standard BODE^3^ analyses; 0% and 6% discounting, no unrelated health system costs, no pYLDs |
| Equity analyses | 1. Equity scenario analyses, firstly, set Māori life expectancy and morbidity (pYLDs) to be the same as non-Māori (as per BODE^3^ protocol) (equitable healthy life expectancy); and secondly, set the screening coverage for Māori to the same as non-Māori (equitable treatment). |
| Coverage | 1. We ran a scenario that was less optimistic with coverage of 45% in Māori and 58% in non-Māori parallel with per the first year of the colorectal screening pilot program. |
| Follow-up | 1. Follow-up for 15 years (rather than over a lifetime). |
| Age varying effect | 1. We did a scenario analysis where 25-40 year olds benefited from an effect size of RR of 0.50 instead of 0.64. |
| Improved eradication | 1. Changing *H. pylori* treatment to include Levofloxacin instead of clarithromycin is likely to have a greater eradication rate. In this scenario we modeled a 90% eradication rate for Māori and non-Māori,[20] such as that achieved by a 10-14 days course of levofloxacin based triple therapy.[21] |
| Retest excluded | 1. The fecal antigen retest step was excluded. |

#### Fecal antigen probabilistic sensitivity analysis

For the fecal antigen scenario the effect size and the costs were adjusted to reflect differences between the tests, including the greater sensitivity of the fecal antigen test in detecting *H. pylori*.

The cost was adjusted to reflect, firstly, the greater laboratory costs with fecal antigen testing and, secondly, the lower detection rate of fecal antigen compared to serology, based on a screening study from Japan with an 8% lower detection rate by fecal antigen compared to serology (56.4%/61.4%).[22]

Table S6: Inputs to the Markov model for the fecal antigen scenarios

| Fecal antigen scenario | | | Māori | Non-Māori |
| --- | --- | --- | --- | --- |
|  | Fecal antigen detection of *H. pylori* as a function of seroprevalence[23] | | | |
|  |  | Sensitivity of the fecal antigen test(${Se}_{F.Ag}$)[24] | 0.95 [0.94-0.96] | |
|  |  | Positive test rate of fecal antigen compared to serology[22] | 0.919 | |
|  | Cost per person invited (fixed costs) (± 20%) | | $43.60 (± 20%) | |
|  | Cost per person tested (test and results) | | $89.17 (± 20%) | |
|  | Cost per person with a positive test (GP visit, treatment, retest, complications) | | $177.30 (± 20%) | |
|  | Cost per person where eradication failed (GP visit, treatment, complications) | | $129.85 (± 20%) | |

## Further results

Table S7: Values for the data points in Figure two: modeled cost-effectiveness of a *H. pylori* screening program in New Zealand by ethnicity, sex and age for the 25-69 year old population in 2011, expected values (deterministic analysis), ICER and costs are in NZD 2011

|  |  |  | Age group (years) | |  |  |  |  |  |  |  |
| --- | --- | --- | --- | --- | --- | --- | --- | --- | --- | --- | --- |
| Ethnicity | Sex |  | 25-29 | 30-34 | 35-39 | 40-44 | 45-49 | 50-54 | 55-59 | 60-64 | 65-69 |
| Non-Māori | Male | IncCost | 99.88831 | 104.232- | 109.0691 | 114.5879 | 120.5389 | 126.9349 | 133.5381 | 139.2548 | 143.6876 |
|  |  | IncQALY | 0.002762 | 0.003476 | 0.004300 | 0.005245 | 0.006247 | 0.007249 | 0.008077 | 0.008486 | 0.008256 |
|  |  | ICER | 36162.24 | 29987.54 | 25364.29 | 21845.84 | 19295.30 | 17511.81 | 16532.25 | 16409.96 | 17403.65 |
|  | Female | IncCost | 97.16595 | 100.8582 | 104.9090 | 109.4486 | 114.2715 | 119.4755 | 124.8493 | 129.8012 | 134.2626 |
|  |  | IncQALY | 0.001809 | 0.002258 | 0.002757 | 0.003311 | 0.003863 | 0.004351 | 0.004793 | 0.005106 | 0.005136 |
|  |  | ICER | 53699.33 | 44662.59 | 38052.15 | 33051.94 | 29579.08 | 27459.70 | 26046.70 | 25423.30 | 26141.08 |
| Māori | Male | IncCost | 109.9890 | 119.9784 | 131.2833 | 143.9376 | 157.3356 | 171.5308 | 185.9021 | 197.0355 | 205.7835 |
|  |  | IncQALY | 0.010239 | 0.012370 | 0.014432 | 0.016420 | 0.018036 | 0.019316 | 0.019889 | 0.019346 | 0.017617 |
|  |  | ICER | 10742.06 | 9698.811 | 9096.811 | 8766.096 | 8723.372 | 8880.265 | 9346.821 | 10184.79 | 11680.77 |
|  | Female | IncCost | 97.64167 | 105.7223 | 115.3123 | 126.1262 | 137.6869 | 150.2525 | 162.9970 | 173.4296 | 182.8754 |
|  |  | IncQALY | 0.008656 | 0.010150 | 0.011427 | 0.012569 | 0.013311 | 0.013557 | 0.013676 | 0.013528 | 0.012841 |
|  |  | ICER | 11280.85 | 10416.49 | 10091.64 | 10034.66 | 10344.21 | 11083.19 | 11918.76 | 12819.66 | 14241.35 |

Abbreviations: incremental Cost (IncCost), incremental quality adjusted life years (IncQALY), incremental cost-effectiveness ratio (ICER)

Table S8: Estimated costs that might be offset (and the resulting change in the Incremental Cost Effectiveness Ratio, ICER) if there was a 25% reduction in dyspepsia among *H. pylori* infected individuals after eradication therapy such as there was in a UK study (USD 117 = NZD 175, which is NZD 153 if spread over ten years and discounted by 3% pa) [25], applied here to 45-49 year olds in the New Zealand as an additional cost offset in the *H. pylori* screening cost-utility model

| Population group (aged 45-49 years) | Cost offset in UK study if *H. pylori* +ve (40-49yrs) [25] | Screening coverage | *H. pylori* seroprevalence | Cost offset per person screened (NZ$) | Incremental cost (NZ$) | Incremental QALYs | ICER adjusted (NZ$) | ICER original (NZ$) | % decrease in ICER |
| --- | --- | --- | --- | --- | --- | --- | --- | --- | --- |
| Māori | $153 | 81% | 29% | $35.00 | $148 | 0.0157 | $7,120 | $9,410 | 24% |
| European/Other | $153 | 84% | 18% | $23.20 | $117 | 0.00506 | $18,600 | $23,200 | 20% |

Note: This simplistic analysis does not include the QALY gain from reduced dyspepsia

## References

1. Lansdorp-Vogelaar I, Sharp L: **Cost-effectiveness of screening and treating Helicobacter pylori for gastric cancer prevention**. *Best Practice Research Clinical Gastroenterology* 2013, **27**(6):933-947.

2. International Agency for Research on Cancer, World Health Organization: ***Helicobacter pylori* Eradication as a Strategy for Preventing Gastric Cancer**. In: *Working Group Report.* vol. 8. Lyon: International Agency for Research on Cancer; 2014.

3. Parsonnet J, Harris RA, Hack HM, Owens DK: **Modelling cost-effectiveness of Helicobacter pylori screening to prevent gastric cancer: a mandate for clinical trials**. *The Lancet* 1996, **348**(9021):150-154.

4. Harris RA, Owens DK, Witherell H, Parsonnet J: **Helicobacter pylori and Gastric Cancer: What Are the Benefits of Screening Only for the CagA Phenotype of H. pylori?** *Helicobacter* 1999, **4**(2).

5. Fendrick AM, Chernew ME, Hirth RA, Bloom BS, Bandekar RR, Scheiman JM: **Clinical and Economic Effects of Population-Based Helicobacter pylori Screening to Prevent Gastric Cancer**. *Archives of Internal Medicine* 1999, **159**:142-148.

6. Mason J, Axon ATR, Forman D, Duffett S, Drummond M, Crocombe W, Feltbower R, Mason S, Brown J, Moayyedi P: **The cost-effectiveness of population Helicobacter pylori screening and treatment: a Markov model using economic data from a randomised trial**. *Alimentary pharmacology & therapeutics* 2002, **16**:559-568.

7. Roderick P, Davies R, Raftery J, Crabbe D, Pearce R, Bhandari P, Patel P: **The cost-effectiveness of screening for Helicobacter pylori to reduce mortality and morbidity from gastric cancer and peptic ulcer disease: a discrete-event simulation model**. *Health Technology Assessment* 2003, **7**(6).

8. Lee YC, Lin JT, Wu HM, Liu TY, Yen MF, Chiu HM, Wang HP, Wu MS, Hsiu-Hsi Chen T: **Cost-effectiveness analysis between primary and secondary preventive strategies for gastric cancer**. *Cancer Epidemiol Biomarkers Prev* 2007, **16**(5):875-885.

9. Xie F, Luo N, Lee H-P: **Cost effectiveness analysis of population-based serology screening and 13 C-Urea breath test for *Helicobacter pylori* to prevent gastric cancer: A markov model**. *World Journal of Gastroenterology* 2008, **14**(19):3021-3027.

10. Xie F, Luo N, Blackhouse G, Goeree R, Lee HP: **Cost-effectiveness analysis of Helicobacter pylori screening in prevention of gastric cancer in Chinese**. *International Journal of Technology Assessment in Health Care* 2008, **24**(1):87-95.

11. Yeh JM, Kuntz KM, Ezzati M, Goldie SJ: **Exploring the cost-effectiveness of Helicobacter pylori screening to prevent gastric cancer in China in anticipation of clinical trial results**. *International Journal of Cancer* 2009, **124**(1):157-166.

12. Xie F, O'Reilly D, Ferrusi IL, Blackhouse G, Bowen JM, Tarride JE, Goeree R: **Illustrating economic evaluation of diagnostic technologies: comparing Helicobacter pylori screening strategies in prevention of gastric cancer in Canada**. *Journal of the American College of Radiology* 2009, **6**(5):317-323.

13. Cheng HC, Wang JD, Chen WY, Chen CW, Chang SC, Sheu BS: **Helicobacter pylori test-and-treat program can be cost-effective to prevent gastric cancer in Taiwanese adults: referred to the nationwide reimbursement database**. *Helicobacter* 2015, **20**(2):114-124.

14. **Trendly Beta: Promoting High Performance in Health** [<http://trendly.co.nz/Home/DHBIndicatorSummaryReport>]

15. McDonald AM, Sarfati D, Baker MG, Blakely T: **Trends in Helicobacter pylori infection among Maori, Pacific, and European Birth cohorts in New Zealand**. *Helicobacter* 2015, **20**(2):139-145.

16. Hsiang J, Selvaratnam S, Taylor S, Yeoh J, Tan Y-M, Huang J, Patrick A: **Increasing primary antibiotic resistance and ethnic differences in eradication rates of Helicobacter pylori infection in New Zealand—a new look at an old enemy**. *New Zealand Medical Journal* 2013, **126**(1384):64-76.

17. Foster R, Blakely T, Wilson N, O’Dea D: **Protocol for Direct Costing of Health Sector Interventions for Economic Modelling (Including Event Pathways)**. In: *Public Health Monograph Series No 27.* Edited by Burden of Disease Epidemiology Equity and Cost-Effectiveness Programme (BODE³). Wellington: University of Otago, Wellington; 2013.

18. Pharmaceutical Management Agency: **New Zealand Pharmaceutical Schedule**. In*.*: New Zealand Government; 2015.

19. Foster R, Preval N: **Costing of pharmaceuticals in New Zealand for health economic studies: backgrounder and protocol for costing**. In: *Public Health Monograph Series No 20.* Edited by Burden of Disease Epidemiology Equity and Cost-Effectiveness Programme (BODE³). Wellington: University of Otago, Wellington; 2011.

20. Fraser A: **Treatment for Helicobacter infection in New Zealand: A discussion document**. In*.* Edited by Gastroenterology NZSo: New Zealand Society of Gastroenterology; 2013.

21. Li BZ, Threapleton DE, Wang JY, Xu JM, Yuan JQ, Zhang C, Li P, Ye QL, Guo B, Mao C *et al*: **Comparative effectiveness and tolerance of treatments for Helicobacter pylori: systematic review and network meta-analysis**. *BMJ* 2015, **351**:h4052.

22. Shimoyama T, Oyama T, Matsuzaka M, Danjo K, Nakaji S, Fukuda S: **Comparison of a Stool Antigen Test and Serology for the Diagnosis of Helicobacter pylori Infection in Mass Survey**. *Helicobacter* 2009, **14**:87-90.

23. **The changing face of Helicobacter pylori testing** [<http://www.bpac.org.nz/BT/2014/May/h-pylori.aspx>]

24. Gisbert JP, de la Morena F, Abraira V: **Accuracy of monoclonal stool antigen test for the diagnosis of H. pylori infection: a systematic review and meta-analysis**. *The American journal of gastroenterology* 2006, **101**(8):1921-1930.

25. Ford AC, Forman D, Bailey AG, Axon AT, Moayyedi P: **A community screening program for Helicobacter pylori saves money: 10-year follow-up of a randomized controlled trial**. *Gastroenterology* 2005, **129**(6):1910-1917.
